# New methods for modelling EQ‐5D‐5L value sets: An application to English data

**DOI:** 10.1002/hec.3560

**Published:** 2017-08-18

**Authors:** Yan Feng, Nancy J. Devlin, Koonal K. Shah, Brendan Mulhern, Ben van Hout

**Affiliations:** ^1^ Office of Health Economics London UK; ^2^ School of Health and Related Research University of Sheffield Sheffield UK; ^3^ Centre for Health Economics Research and Evaluation University of Technology Sydney Sydney NSW Australia

**Keywords:** econometric modelling, EQ‐5D‐5L, health utilities, health‐related quality of life, value set

## Abstract

Value sets for the EQ‐5D‐5L are required to facilitate its use in estimating quality‐adjusted life years. An international protocol has been developed to guide the collection of stated preference data for this purpose and has been used to generate EQ‐5D‐5L valuation data for England. The aim of this paper is report the innovative methods used for modelling those data to obtain a value set.

Nine hundred and ninety‐six members of the English general public completed time trade‐off (TTO) and discrete choice experiment (DCE) tasks. We estimate models, with and without interactions, using DCE data only, TTO data only, and TTO/DCE data combined. TTO data are interpreted as both left and right censored. Heteroskedasticity and preference heterogeneity between individuals are accounted for. We use Bayesian methods in the econometric analysis. The final model is chosen based on the deviance information criterion (DIC).

Censoring and taking account of heteroskedasticity have important effects on parameter estimation. For DCE data only, TTO data only, and DCE/TTO data combined, models with parameters for all dimensions and levels perform best, as judged by the DIC. Taking account of heterogeneity improves fit, and the multinomial model reports the lowest DIC. This paper presents approaches that suit observed characteristics of EQ‐5D‐5L valuation data and recognise respondents' preference heterogeneity. The methods described are potentially relevant to other value set studies.

## 1 INTRODUCTION

Generic preference‐based measures of health‐related quality of life (HRQoL) have been developed primarily for use in the economic evaluation of health care technologies (Brazier, Ratcliffe, Salomon, & Tsuchiya, 2017). The EQ‐5D is the most well‐known and widely used generic preference‐based measure (Devlin & Krabbe, 2013), with applications in clinical studies, reimbursement decision making, health care monitoring, and population health studies. It comprises five dimensions: mobility, self‐care, usual activities, pain/discomfort, and anxiety/depression. In the original version of the instrument, each dimension has three severity levels: no, some, or extreme problems. In order to increase the instrument's sensitivity to changes in health, a new version of the instrument with five levels on each of the five dimensions, that is, the EQ‐5D‐5L, has been developed (Herdman et al., 2011).

To generate country‐specific EQ‐5D value sets, general public respondents are asked to value a subset of health states described by the instrument. A number of different techniques can be used to obtain these values, such as standard gamble, time trade‐off (TTO), or visual analogue scale. They may also be derived indirectly using the discrete choice experiment (DCE) method, where values on a latent scale are derived from health state comparisons. To value the EQ‐5D‐5L in England we collected data from 996 individuals following a protocol developed by the EuroQol Group (Oppe, Devlin, van Hout, Krabbe, & de Charro, 2014), which comprises a combination of TTO and DCE tasks.

Van Hout and McDonnell (1992) presented the first EQ‐5D value function, later published by van Busschbach, McDonnell, Essink‐Bot, and van Hout (1999). Regression techniques were used to estimate the coefficients for each level and dimension, which could then be used to generate values for all the health states described by the instrument. A number of issues related to the modelling approaches used to develop value sets that were relevant at that time are just as important now. Some of the issues are technical in nature, some are related to specific aspects of the valuation tasks, and some challenge the assumptions made in the valuation studies. An example of the latter is a question about whether the mean should be used as the measure of central tendency when analysing health state values (Devlin, Shah, & Buckingham, 2017).

The primary aim of this paper is to give a detailed report of the methods for modelling the English EQ‐5D‐5L value set, these being quite different from earlier methods. The methods may also be relevant for modelling valuation data for different versions of the EQ‐5D instrument, other countries' EQ‐5D valuation data, and valuation data for other health outcome measures.

The remainder of this paper starts by describing the data collection procedure, exclusion criteria, and approaches to interpreting the data. We then describe a variety of models tested: those that use DCE data only; those that use TTO data only; and those that combine the TTO and DCE data. Special attention is paid to the error distribution in the TTO model, acknowledging the limited range of the data, the fact that the data are actually in intervals, the fact that the variance increases with worsening health states, and preference heterogeneity. We also discuss the criteria used to select the “best” model. Findings are presented in Section 4, including modelling results from the various specifications as well as the sensitivity analysis. The final section discusses the improvements in econometric modelling methods developed in this study and compares these with methods used previously.

## 2 DATA

In 2013, the EuroQol Valuation Technology (EQ‐VT)—computer‐assisted personal interview software—was developed by the EuroQol Group together with a protocol for the collection of EQ‐5D‐5L valuation data using TTO and DCE tasks (Oppe et al., 2014). For the TTO tasks, a composite approach (Janssen, Oppe, Versteegh, & Stolk, 2013) was followed using “conventional” TTO for health states considered better than dead and “lead time TTO” for health states considered worse than dead (Devlin, Buckingham, et al., 2013). Screenshots for the composite TTO and DCE tasks in the EQ‐VT are presented in Oppe et al. (2014). A number of country‐specific value sets for the EQ‐5D‐5L are available: Netherlands (Versteegh et al., 2016), Canada (Xie et al., 2016), Korea (Kim et al., 2016), Uruguay (Augustovski et al., 2016), and Japan (Ikeda et al., 2015). These studies used only TTO data in the econometric modelling to produce the final value sets.

### 2.1 Sampling

Primary data collection was carried out in England by the market research company Ipsos MORI. The valuation data were collected via face‐to‐face interviews in respondents' homes by 48 trained interviewers. A sample of 2,020 addresses from 66 primary sampling units (based on postcode sectors) across England was randomly selected, using the Post Office small user Postcode Address File as the sampling frame. The sample was intended to be representative of adults aged 18 years and over living in private residential accommodation in England. One thousand and four individuals were interviewed between November 2012 and May 2013, with 996 completing the valuation tasks in full.

### 2.2 Study design

Eighty‐six health states were valued using TTO. These were allocated to 10 blocks with 10 health states in each. Each block included the worst health state in the EQ‐5D‐5L descriptive system (55555) and one of the least severe health states. For the DCE tasks, 196 pairs of EQ‐5D‐5L health states were selected and organised into 28 blocks with seven pairs in each. The selection of the health states is reported by Oppe and van Hout (2010). Each respondent completed 10 TTO and 7 DCE tasks. Devlin, Shah, Feng, Mulhern, and van Hout (2017) provide detailed descriptions of the preference elicitation methods used.

### 2.3 Exclusion criteria

In the TTO tasks, 84 respondents were excluded because we judged their valuation data to be implausible. These include 23 respondents who gave the same TTO value for all 10 states and 61 respondents who gave 55555 a value no lower than the value they gave to the mildest health state in their block. This was considered by the study team to represent a “clear inconsistency.” See Engel, Bansback, Bryan, Doyle‐Waters, and Whitehurst (2016) for a review of exclusion criteria applied in national valuation studies. The exclusions were applied to the raw data before any econometric analyses were undertaken. They were not used to define the prior probability distributions in the Bayesian regression analyses.

### 2.4 Final data set

The final TTO data set includes 912 respondents with 9,120 TTO observations. Summary statistics for the TTO values for the 86 health states are reported in Table 1.

**Table 1:** Summary statistics for the 86 TTO health states (*N* = 9,120)

Health state	*N*	Mean	*SD*	Median	Min	Max	Health state	*N*	Mean	*SD*	Median	Min	Max
11112	182	0.85	0.23	0.95	−0.65	1	22434	93	0.53	0.48	0.60	−1	1
11121	181	0.89	0.19	0.95	−0.20	1	23514	80	0.40	0.46	0.50	−1	1
11211	173	0.89	0.18	0.95	0	1	24342	99	0.36	0.51	0.50	−1	1
12111	184	0.87	0.21	0.95	0	1	31524	83	0.45	0.47	0.50	−1	1
21111	192	0.89	0.17	0.95	0	1	52215	80	0.35	0.48	0.50	−1	1
11122	91	0.79	0.28	0.90	0	1	52431	83	0.54	0.41	0.60	−1	1
11212	85	0.82	0.25	0.95	0	1	53412	99	0.44	0.46	0.50	−1	1
11221	93	0.84	0.22	0.95	0	1	54231	93	0.40	0.48	0.50	−1	1
12112	85	0.81	0.26	0.95	0	1	31525	107	0.43	0.46	0.50	−1	1
12121	80	0.81	0.29	0.90	−1	1	32443	80	0.29	0.49	0.50	−1	0.95
21112	74	0.83	0.22	0.90	0	1	33253	99	0.40	0.45	0.50	−1	1
11421	107	0.65	0.38	0.80	−1	1	35143	107	0.27	0.55	0.50	−1	1
13122	93	0.81	0.22	0.90	0.10	1	35332	93	0.59	0.39	0.70	−1	1
14113	83	0.69	0.33	0.80	−0.90	1	43315	83	0.42	0.44	0.50	−0.95	1
11414	107	0.41	0.53	0.50	−1	1	44125	74	0.32	0.51	0.50	−1	1
13313	107	0.69	0.33	0.75	−1	1	45133	80	0.36	0.51	0.50	−1	1
11235	93	0.53	0.42	0.50	−0.95	1	51451	93	0.26	0.45	0.35	−1	1
12513	74	0.61	0.42	0.80	−1	1	24443	83	0.33	0.47	0.40	−1	1
13224	91	0.49	0.48	0.60	−1	1	34244	85	0.26	0.48	0.30	−1	1
21315	83	0.54	0.46	0.60	−1	1	43514	85	0.36	0.53	0.50	−1	1
25122	107	0.52	0.48	0.60	−1	1	45233	107	0.33	0.52	0.50	−1	1
42321	91	0.54	0.44	0.70	−1	1	45413	93	0.34	0.57	0.50	−1	1
11425	93	0.53	0.48	0.60	−1	1	53243	107	0.23	0.58	0.30	−1	1
12244	107	0.32	0.51	0.50	−1	1	34155	80	0.24	0.52	0.35	−1	1
12334	99	0.44	0.49	0.50	−1	1	34515	93	0.32	0.55	0.50	−1	1
12514	93	0.44	0.48	0.50	−1	1	43542	80	0.23	0.45	0.30	−1	0.95
15151	83	0.42	0.45	0.50	−1	1	45144	93	0.17	0.44	0.20	−1	1
21334	99	0.50	0.43	0.60	−1	1	52335	91	0.33	0.51	0.50	−1	1
23152	85	0.39	0.43	0.50	−1	1	53244	107	0.12	0.53	0.10	−1	1
23242	99	0.44	0.47	0.50	−1	1	54153	83	0.27	0.48	0.40	−1	1
25222	107	0.59	0.38	0.65	−1	1	54342	74	0.18	0.55	0.30	−1	1
32314	99	0.51	0.46	0.50	−1	1	55233	107	0.28	0.58	0.50	−1	1
35311	91	0.51	0.50	0.60	−1	1	14554	74	0.15	0.52	0.10	−1	1
42115	93	0.41	0.48	0.50	−1	1	24445	91	0.16	0.57	0.30	−1	1
53221	74	0.58	0.42	0.70	−1	1	24553	93	0.33	0.50	0.50	−1	1
12344	74	0.25	0.50	0.33	−1	1	35245	93	0.18	0.48	0.30	−1	1
25331	107	0.53	0.52	0.70	−1	1	55225	99	0.17	0.57	0.20	−1	1
31514	107	0.39	0.53	0.50	−1	1	44345	74	0.21	0.50	0.25	−1	1
34232	91	0.55	0.44	0.60	−1	1	55424	85	0.25	0.53	0.35	−1	1
51152	93	0.35	0.55	0.50	−1	1	44553	85	0.09	0.54	0.10	−1	0.95
12543	80	0.32	0.52	0.50	−1	1	52455	107	0.07	0.57	0.10	−1	1
21345	85	0.43	0.47	0.50	−1	1	43555	91	0.06	0.59	0.10	−1	1
21444	107	0.15	0.52	0.30	−1	1	55555	912	−0.08	0.52	0.00	−1	0.95

**Figure 1:**
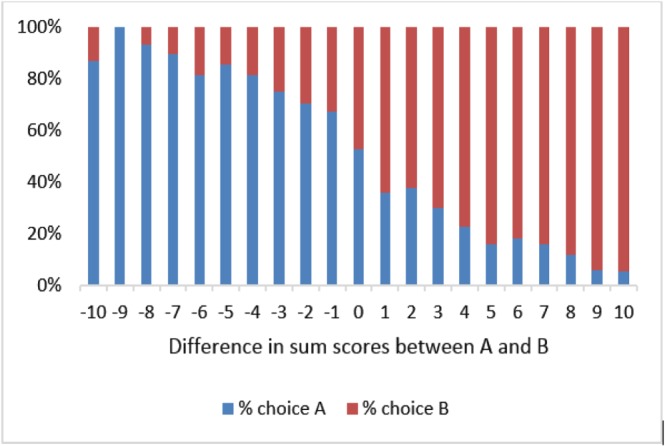
Percentage choosing A or B in the discrete choice experiment tasks versus relative severities of A and B (*N* = 996) [Colour figure can be viewed at wileyonlinelibrary.com]

The final DCE data set includes 996 respondents with 6,972 observations. Each task involved a choice between two health states, labelled “A” and “B.” Among the 996 respondents, five respondents always chose A and five always chose B. For each health state, a “level sum score”—sum of the levels of the five dimensions; a proxy for severity ranging from 5 (for 11111) to 25 (for 55555)—can be calculated. Figure 1 shows the percentage of respondents who chose A, plotted against the differences in the level sum score between the two options.

### 2.5 Interpretation of values at −1, 0, and 1

#### 2.5.1 Censoring at −1

When respondents completing a TTO task value health state “x” as worse than dead, they may, at the extreme, prefer to die now than to live for 10 years in full health (the lead time) followed by 10 years in “x.” In that case the resultant value, given the variant of TTO used in the EQ‐5D‐5L valuation protocol, is −1. However, we cannot exclude the possibility that respondents who respond in this way would have traded more time in full health had they been presented with a longer lead time, in which case their value would be lower than −1 (Devlin, et al., 2013). As such, when a value of −1 is observed it can be interpreted as −1 *or lower*, which makes these values, in a statistical sense, “left censored” at −1.

#### 2.5.2 Censoring at 0

When the TTO data for each respondent were plotted against the predicted TTO values from the 10‐parameter DCE tariff, we found that most respondents' data followed a negative gradient as expected (i.e., valuing more severe health states lower than less severe health states). However, some respondents use zero as the minimum value more than once, including when valuing the worst health state 55555. This suggests that those respondents did not want to go below zero (e.g., do not believe there is such a thing as a health state so bad that experiencing it for 10 years would be worse than dead). Consequently, these respondents do not distinguish between 55555 and other health states that are logically better than 55555, leading to a situation in which no value is attached to improvements from 55555 to less severe health states. Our interpretation is that when a respondent valued more than one health state (almost always including 55555) at zero, these values are not necessarily equal. This is captured by interpreting the observed zero values as being either zero or less than zero. In statistical terms, those zeros are interpreted as being “censored at zero.” In the analysis reported here, this concerns 150 respondents and 595 observed zero values.

Further, a number of respondents valued 55555 at zero whilst valuing more than one other health state at less than zero. This is logically inconsistent. We censored those negative values and associated zero values at zero. This concerns 27 individuals and 154 observations.

**Figure 2:**
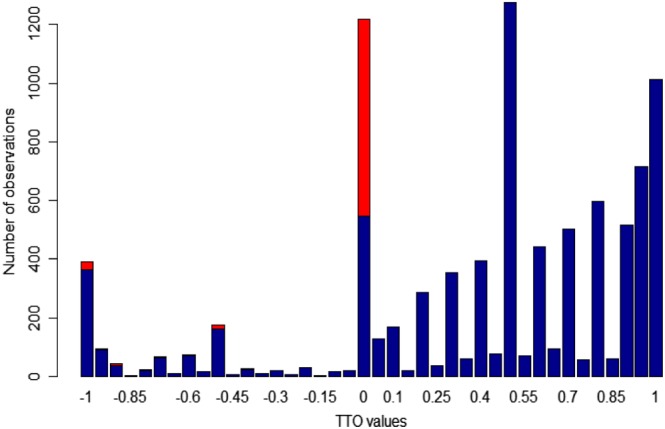
Number of observations censored and not censored at zero against the time trade‐off (TTO) value [Colour figure can be viewed at wileyonlinelibrary.com]

Figure 2 shows the number of observations for each TTO value. The red bars show the number of observations censored at zero. In total, we censored 749 observations (595 + 154 = 749) at zero.

#### 2.5.3 Censoring at 1

Some respondents gave relatively low values, for example, 0.5 or 0, to the least severe states resulting in relatively large differences between the mean and the median value. For example, the median and mean for health state 11211 are 0.95 and 0.89, respectively. The distribution of observed values is the result of a distribution of true values (different people may have different values) and errors (each observed value may not be the true value). Although one can make an error to the left of the scale and value this health state at 0, one cannot make an equivalent error to the right of the scale and value it at, say, 2. As a result, the distribution is likely *not* to be normally distributed, which also explains why the mean and median are quite different. Now, although the distribution of true values is “truncated” at 1, the distribution of true values plus errors may be seen as “censored” at 1. In combination, values at 1 could be considered as being either 1 or greater than 1 (i.e., “right censored”) and this is how we treat all values at 1. This may be considered arbitrary; however, we feel that this fits the data generating process better than to assume that the errors follow a normal distribution (without taking any account of truncation or censoring), as is traditionally done.

### 2.6 Weighting

Our sample generally reflects the distribution of the English population in terms of key sociodemographic characteristics (Appendix S1). However, there is a noticeable underrepresentation of individuals aged between 18 and 29 years old. To adjust our sample to reflect the true distribution of the population in England, we apply a weight for each age band (Office for National Statistics, 2016). Our sample is divided into eight age bands, that is, 18–29 years, 30–39 years, 40–49 years, 50–59 years, 60–69 years, 70–79 years, 80–89 years, and 90 years and above. The weight for each band is calculated as the ratio of the proportion of the general population in England/Wales in that band to the corresponding proportion in our sample.

All models in the econometric analyses applied these weights to adjust the distribution of the sample.

## 3 METHODS

Both TTO and DCE data can be used individually to produce a value set. We present results using DCE data, TTO data, and TTO/DCE data combinations.

### 3.1 Model parameter specification

Within each method, models were estimated with 5, 9, 10, and 20 parameters, with and without interaction terms, and with and without terms capturing some degree of decreasing marginal severity, corresponding to the “N3 term” used in the UK 3‐level EQ‐5D tariff (Dolan, 1997).

The five‐parameter model estimates one parameter for each dimension. The level descriptors (no, slight, moderate, severe, and extreme/unable problems) are captured by five numbers, that is, 0, 1, 2, 3, and 4, respectively. The assumption behind this model is that there is a linear relationship between the TTO values and the five dimensions. Within each dimension, the utility decrements for moderate problems are assumed to be twice as large as for slight problems, the utility decrements for severe problems are assumed to be three times as large as for slight problems, and so forth.

The nine‐parameter model estimates one parameter per dimension and one parameter per level (4 levels + 5 dimensions = 9 parameters). In theory, the five dimension parameters could add up to one. 1The estimated parameter for the anxiety/depression dimension (*β*_*ad*_) is formalised as a function of the parameters for the other four dimensions (*β*_*mo*_,*β*_*sc*_,*β*_*ua*_,*β*_*pd*_) with *β*_*ad*_ = 1 − *β*_*mo*_−*β*_*sc*_−*β*_*ua*_−*β*_*pd*_.
 To save the degrees of freedom, we actually estimate four dimension parameters rather than five. Therefore, the nine‐parameter model could be viewed as an eight‐parameter model (4 levels + 4 dimensions = 8 parameters) with the additional dimension parameter constrained. The assumptions behind this model is that there is a linear relationship between the TTO values and the five dimensions. The impact of each level is the same across all five dimensions.

Unlike the nine‐parameter model, the 10‐parameter model estimates two parameters for Level 5 (one for the mobility, self‐care, and usual activity dimensions where Level 5 describes being “unable” to do a certain function; the other for the pain/discomfort and anxiety/depression dimensions where Level 5 described having “extreme” problems). As in the nine‐parameter model, we estimate four dimension parameters. Therefore, we actually estimate nine parameters with one dimension parameter constrained instead of 10 parameters.

The 20‐parameter model estimates four parameters for each dimension and one parameter per level, with the “no problems” level used as the baseline (4 levels × 5 dimensions = 20 parameters). This model allows the coefficients to differ between dimensions, and for the importance of each level of problems to differ between dimensions.

Below, we use the 20‐parameter model without interactions to illustrate our methods.

### 3.2 Modelling the discrete choice data

When considering the DCE data, respondents compare the utilities of two health states, that is, *V*_*ijl*_ and *V*_*ijr*_. The term *V*_*ijl*_ (*V*_*ijr*_) refers to the utility gained individual *i* for health state *x*_*kijl*_ (*x*_*kijr*_) presented on the left (right) hand side within DCE pair *j*—see Equation 1.

(1) $$V_{\mathrm{ijl}}=\alpha -\sum_{k=1}^{20}\beta_{k}x_{k}^{\mathrm{ijl}}+e_{i}^{\mathrm{lj}}\;<?>\;V_{\mathrm{ijr}}=\alpha -\sum_{k=1}^{20}\beta_{k}x_{k}^{\mathrm{ijr}}+e_{i}^{\mathrm{rj}}.\;$$

The variable *x* represents the health state by 20 dummy variables. The first four dummies refer to the mobility dimension and take the value 1 if the description of mobility is with “slight,” “moderate,” “severe,” or “extreme/unable” problems. The second four dummies (5 to 8) relate to the self‐care dimension, the third four dummies (9 to 12) relate to the usual activities dimension, the fourth four dummies (13 to 16) relate to the pain/discomfort dimension, and the fifth four dummies (17 to 20) relate to the anxiety/depression dimension. The parameter *α* reflects the value for full health. *β* is a 20 × 1 vector of which the first four elements reflect the disutility associated with being in either “mild,” “moderate,” “severe,” or “extreme” problems in mobility. The fifth to the eighth elements reflect the disutilities in the different levels of self‐care, and so on such that the 17th to 20th elements reflecting the disutility considering anxiety/depression. $e_{i}^{j}$ refers to the error term which we assume follows either an extreme value distribution (as in logistic regression) or a normal distribution (as in probit analyses). Both *V*_*ijl*_ and *V*_*ijr*_ are unobserved latent variables imagined to represent the value attached to health states. The relationship between the utilities of the two health states in a pair is unknown (?). It is assumed that respondents choose the left hand side health state in DCE pair *j* when the utility is larger (>) than that of the right side health state (*V*_*ijl*_ > *V*_*ijr*_). The right hand side health state is chosen when the utility is smaller (<) than that of the right side health state (*V*_*ijl*_ < *V*_*ijr*_). The value of *α* is assumed to be cancelled out and therefore cannot be estimated.

### 3.3 Modelling the TTO data

When modelling the DCE data, the parameters only reflect relative values. The data provide information on the relative preference of one health state over another. When using TTO data, the parameters can be interpreted as measuring a deviation from full health on a scale anchored at 1 (representing full health) and 0 (the value for dead). As with the DCE data, we assume a TTO value function that is linear between the TTO value and the description of the health state. The specification is shown by Equation 2.

(2) $$V_{i}^{j}=1-\left(\sum_{k=1}^{20}\beta_{k}x_{k}^{\mathrm{ij}}\right)+e_{i}^{j}.\;$$

$V_{i}^{j}$ is the TTO value for health state *j* for respondent *i*. Parameter *β*_*k*_ reflects the decrement from full health for the health state described by *x*_*k*_. The error term $e_{i}^{j}$ measures the difference between the observed TTO value and the mean value. It captures random errors as well as differences of opinion between respondents about health states. In a linear regression analysis, the random error term is assumed to follow a normal distribution with a mean of zero and constant variance. We follow this assumption but, as indicated above, there may be censoring at −1, 0, and 1.

Three further issues require consideration. First, it is observed that the variance of TTO values is larger for more severe health states than for less severe health states. This is due to a divergence in preferences regarding these states, but also increased respondent error. Second, there are only 41 unique TTO values between −1 and 1 available to respondents. Third, we limit the parameter space such that coefficients are always logically consistent.

#### 3.3.1 Heteroskedasticity/heterogeneity

The variation of TTO values between more severe and less severe states means that the error terms in modelling the TTO data show heteroskedasticity. One explanation is that values for the mildest health states could sensibly be in a relative narrow range of 0.8 and 1 (for example), whereas the sensible range of values for the more severe health states could be much larger, for example, between −1 and 0.5. This is because respondents could use 1 as a baseline to value the mildest health states. However, for severe health states, respondents apply their own scale and there is no baseline value to use. They can provide any value between −1 to 1. Furthermore, this might be explained by general public respondents' hypothetical bias (Burström et al., 2014; Leidl & Reitmeir, 2011) as they were asked to value hypothetical health states that in many cases will be more severe than those that they themselves have experienced to date. Respondents may therefore be more likely to report bias in the valuation of severe states than in the valuation of mild states. This heteroskedasticity is captured by applying an exponential relationship between the variance and the mean of the error terms per health state, adding two parameters to the model. A positive relationship between the two variables is expected as it indicates that the respondents use a smaller range of TTO values for mild health states than for severe health states.

An alternative and probably more fundamental way of capturing the increasing variance in the error terms with the increasing level of severity in health states is to take into account the heterogeneity of respondents' opinions. We observe that respondents effectively use different TTO scales, for example, some respondents never score negative TTO values, some score both negative and positive TTO values, and some express “extreme” views about some health states. It is expected that respondents disagree more about severe health states than milder health states. The heterogeneity of TTO scales that respondents used could be explained by disagreement about the value of dead between respondents. This is captured by introducing a parameter for disutility scale *γ*, which may differ between respondents. The specification is reported by Equation 3.

(3) $$V_{i}^{j}=1-\gamma_{i}\left(\sum_{k=1}^{20}\beta_{k}x_{k}^{\mathrm{i j}}\right)+e_{i}^{j}\;$$

We investigate three assumptions of the distribution in *γ*: (a) a normal distribution with mean 1 and variance that follows a gamma distribution; (b) a lognormal distribution with mean 1 and variance that follows a gamma distribution; and (c) a multinomial distribution with probability density on a number of discrete values. It is envisaged that the tail in the lognormal distribution may well reflect the extreme values reported by some respondents. The multinomial model corresponds to the notion that there may be a number of latent groups, each having their own mean and variance. We allowed the number of probability density groups to vary from 2 to 10. For normalisation, the mean value of *γ* for one of the latent groups has its value constrained to 1. In this model, we also assume that there is heteroscedasticity within and between each latent group by allowing the variance of the TTO data for each respondent's age category within and between latent groups to be different.

#### 3.3.2 Continuity of the TTO data

Given the study protocol, respondents can only give 41 distinct values (Oppe et al., 2014). These range from −1 to 1 with steps of 0.05 between each distinct value. Apart from the two boundary values −1 and 1, we assume each observed TTO value x lies within the range [x − 0.025, x + 0.025]. The value 0.025 is the midpoint of the gap between two neighbouring TTO values. For instance, when a respondent gives the value 0.5, it suggests a scale of 0.475 and 0.525, which are the midpoints between 0.5 and the nearest available values 0.45 and 0.55. More subtle rules to define the scale for observed TTO values are possible. Our decision is determined by the EQ‐VT design, in particular regarding the process that respondents follow to arrive at their TTO values. In our case, the midpoint was considered as an appropriate rule for defining the scales for observed values.

We analyse how the standard censored model changes when we treat TTO data points as intervals. The interval censoring triggers the question about how to censor TTO values at −1 and 1. When we observe a TTO value at 1, the observed value suggests a scale of 0.975 and 1. Instead of censoring the TTO value at the top end of 1, we censored it at 0.975 when modelling the TTO data. The same analysis was applied to the bottom end. Therefore, we censored TTO data at −0.975 rather than −1 when modelling the TTO data.

#### 3.3.3 Forcing consistency

Logically consistent estimates are observed when the absolute values for the parameters for higher level problems exceed those for lower level problems. However, when estimating the parameters freely, “logically inconsistent” estimates may be observed. It is hypothesised that although respondents may not always distinguish between different levels, they do not intend to reverse the ordering. The estimated parameters should be logically consistent if respondents are able to correctly distinguish between different levels. The parameter space is used to reflect this. When defining our preferred models, this is captured by first estimating the parameters for the “slight” levels and then estimating those for the more severe levels by subsequently adding quadratic terms (which are non‐negative). 2The estimated parameter for the moderate level in a given dimension d is formulised as the sum of the estimated parameter for the slight level and a quadratic term, that is, *β*_2*d*_ = *β*_1*d*_ + (*r*_1*d*_)^2^; the estimated parameter for the severe level in a given dimension d is formulised as the sum of the estimated parameter for the moderate level and a quadratic term, that is, *β*_3*d*_ = *β*_2*d*_ + (*r*_2*d*_)^2^; the estimated parameter for the extreme/unable level in a given dimension d is formulised as the sum of the estimated parameter for the severe level and a quadratic term, that is, *β*_4*d*_ = *β*_3*d*_ + (*r*_3*d*_)^2^. *r*_1*d*_, *r*_2*d*_, and *r*_3*d*_ are also parameters to be estimated.


### 3.4 Hybrid model

Both the TTO and DCE data provide information about the values of health states. If the same value‐function dictates the answers to both types of question, one would expect the beta coefficients to reflect the same relative weights and as such to be identical up to a linear transformation between the TTO and DCE models. Following a hybrid modelling approach introduced by Rowen, Brazier, and van Hout (2014), we use the TTO and DCE data together in a Bayesian regression assuming that the beta parameters in the DCE model are a linear function of the beta parameters in the TTO model.

### 3.5 Model implementation

All models are fitted in WinBUGS with one chain, each with a burn‐in of 2,000 iterations followed by 7,000 iterations. Convergence was assessed by visual inspection of the autocorrelation graphs and by assessing whether the last 2,000 iterations gave different estimates (± than 0.01) from the average preceding 2,000 iterations.

For the three hybrid models with different assumptions about the distribution in slope, we assume five Normal priors N(0.1, 1) for the Level 2 parameters and wide Normal priors N(0.01, 1) for quadric parameters. Normal priors N(0, 0.1) and N(1, 0.01) are assumed for the constant and slope terms, respectively, that link the TTO and DCE data together.

For the multinomial slope model, we assume Gamma priors Γ(0.1, 0.1) for two slope parameters and the other slope parameter constrained to be one with Gamma prior Γ(1,000, 1,000). The three parameters for the probabilities of being in the three latent groups are assume with Dirichlet priors Dir(0.3, 0.3, 0.4). For the lognormal slope model, we assume the slope ~ lnN(μ, σ^2^) with priors σ^2^ ~ Γ(1,1) and μ = −0.5/σ^2^. For the normal slope model, we assume the slope ~ N(1, σ^2^) with prior σ^2^ ~ Γ(1,1).

### 3.6 Criterion for the “best” model selection

We use the deviance information criterion (DIC) to compare performance of the models within each group. The best models estimated using DCE data only, TTO data only, DCE/TTO data combined and taking account of heteroscedasticity, and DCE/TTO data combined and taking account of heteroscedasticity and heterogeneity were selected. The DIC is also used to compare the performance between the best models from each group. The coefficient ordering and the face validity of the value range were also assessed.

It should be noted that the “best” model is not necessarily expected to predict the mean observed values from the TTO data the best. That is because some observations are censored, and as a result, the mean is not the best measure of central tendency. Furthermore, we use not only the TTO data but also the DCE data in the hybrid model, whereas observed values are available only from the TTO data.

### 3.7 Sensitivity analyses

Five sensitivity analyses are conducted to check the robustness of the 20‐parameter hybrid model results. Each analysis reveals the impact of one potentially arbitrary decision we made. First, we check the impact of exclusion criteria on the modelling results. We run the hybrid model without excluding any TTO observations from the data set. Second, we check the impacts of censoring the TTO data on the modelling results by sequentially (once at a time) removing the TTO data censoring at −1, 0, and 1. Finally, we run the 20‐parameter hybrid model without censoring the TTO data at any data point.

R 3.2.0 and WinBUGS 14 are used for all modelling analyses. The operating system for producing all results was Windows 10 Version 1607 for x64‐based systems. The results were produced on 10th January 2017.

## 4 RESULTS

The results for the 5‐, 9‐, and 10‐parameter models for the DCE, TTO, and combined data sets are reported in Table 2. The five‐parameter model indicates which dimensions get the highest weight, that is, pain/discomfort followed by anxiety/depression. The DCE data indicate a larger weight for mobility than for self‐care and usual activities, whereas the TTO data indicate that self‐care gets the lowest weight of the five dimensions.

**Table 2:** Estimates using the 5‐, 9‐, and 10‐parameter models

	TTO model		DCE model		Hybrid model	
	Coef	*SD*	Coef	*SD*	Coef	*SD*
5‐parameter model
Mobility	0.055	0.004	0.332	0.016	0.061	0.002
Self‐care	0.043	0.004	0.238	0.014	0.044	0.002
Usual activities	0.050	0.004	0.201	0.014	0.043	0.002
Pain/discomfort	0.081	0.004	0.412	0.015	0.080	0.002
Anxiety/depression	0.077	0.004	0.389	0.016	0.077	0.002
DIC	56937.9		7728.2		64655.1	
9‐parameter model						
Mobility	0.195	0.012	0.213	0.007	0.205	0.007
Self‐care	0.159	0.012	0.162	0.007	0.161	0.006
Usual activities	0.148	0.012	0.134	0.007	0.139	0.006
Pain/discomfort	0.255	0.011	0.249	0.008	0.250	0.007
Anxiety/depression	0.244	0.011	0.241	0.007	0.244	0.006
Slight	0.259	0.021	1.475	0.146	0.289	0.015
Moderate	0.472	0.027	1.791	0.143	0.406	0.016
Severe	1.149	0.028	5.342	0.181	1.115	0.019
Unable/extreme	1.181	0.020	6.226	0.191	1.225	0.016
DIC	56860.3		7513.0		64371.5	
10‐parameter model						
Mobility	0.185	0.012	0.213	0.008	0.202	0.006
Self‐care	0.148	0.011	0.164	0.008	0.157	0.006
Usual activities	0.133	0.012	0.135	0.008	0.135	0.007
Pain/discomfort	0.269	0.013	0.248	0.009	0.256	0.007
Anxiety/depression	0.265	0.013	0.240	0.008	0.249	0.007
Slight	0.252	0.020	1.472	0.138	0.287	0.015
Moderate	0.456	0.028	1.792	0.146	0.404	0.017
Severe	1.132	0.029	5.355	0.181	1.108	0.019
Unable	1.368	0.067	6.210	0.244	1.270	0.033
Extreme	1.048	0.043	6.263	0.243	1.188	0.028
DIC	56851.1		7515.4		64370.8	

*Note*. DCE = discrete choice experiment; DIC = deviance information criterion; TTO = time trade‐off.

The nine‐parameter model offers a substantial improvement in the DIC for the DCE data and the TTO data. The decrements from slight to moderate and from severe to unable/extreme are much smaller than the decrements from moderate to severe. Having different parameters for Level 5 based on descriptor (i.e., separating the unable and extreme descriptors) improves the TTO model but not the DCE model. The coefficient for unable is larger than that for severe in all three 10‐parameter models but this is not the case for the coefficient for extreme in the TTO model, which suggests an “inconsistency.”

The results for the 20‐parameter model are presented in Table 3. There is an improvement in the DIC in the 20‐parameter models for estimations that use the TTO data only and the DCE data only in comparison to the corresponding 10‐parameter models. Results from the unrestricted hybrid model are characterised by two logical inconsistencies: one between Levels 4 and 5 on the usual activities dimension and one between Levels 4 and 5 on the anxiety/depression dimension. The inconsistencies are not observed when applying restrictions to limit the parameter space (last column). The DIC improves in the restricted hybrid model, although the coefficients of the restricted and unrestricted hybrid models show little change.

**Table 3:** The 20‐parameter model with and without restriction

	TTO model		DCE model		Unrestricted hybrid model		Restricted hybrid model	
	Coef	*SD*	Coef	*SD*	Coef	*SD*	Coef	*SD*
Mobility								
Slight	0.036	0.015	0.358	0.057	0.060	0.009	0.062	0.008
Moderate	0.079	0.018	0.440	0.066	0.080	0.010	0.080	0.010
Severe	0.202	0.020	1.107	0.067	0.215	0.010	0.213	0.010
Unable	0.246	0.018	1.430	0.074	0.266	0.010	0.266	0.011
Self‐care								
Slight	0.044	0.013	0.240	0.060	0.051	0.009	0.053	0.009
Moderate	0.078	0.018	0.417	0.069	0.084	0.011	0.083	0.010
Severe	0.147	0.018	0.983	0.068	0.181	0.010	0.184	0.010
Unable	0.227	0.017	1.025	0.063	0.208	0.010	0.207	0.009
Usual activities								
Slight	0.051	0.015	0.232	0.059	0.049	0.009	0.051	0.008
Moderate	0.106	0.018	0.226	0.065	0.067	0.010	0.066	0.009
Severe	0.182	0.017	0.784	0.066	0.168	0.010	0.163	0.008
Unable	0.156	0.019	0.816	0.067	0.163	0.010	0.168	0.008
Pain/discomfort								
Slight	0.050	0.012	0.359	0.061	0.058	0.009	0.059	0.009
Moderate	0.067	0.018	0.415	0.068	0.082	0.010	0.080	0.010
Severe	0.283	0.018	1.222	0.070	0.259	0.011	0.258	0.010
Extreme	0.301	0.018	1.613	0.073	0.311	0.011	0.308	0.011
Anxiety/depression								
Slight	0.073	0.013	0.327	0.062	0.068	0.009	0.069	0.009
Moderate	0.126	0.018	0.374	0.068	0.097	0.010	0.094	0.010
Severe	0.313	0.017	1.338	0.070	0.288	0.009	0.282	0.008
Extreme	0.269	0.017	1.454	0.073	0.283	0.010	0.286	0.008
DIC	56842.9		7510.9		64359.4		64102.9	

*Note*. DCE = discrete choice experiment; DIC = deviance information criterion; TTO = time trade‐off.

Table 4 reports results from three 20‐parameter hybrid models with different assumptions of distribution in slope. The last column reports the results from multinomial slope model with three latent groups that divided respondents based on the similarity of their slopes for disutility in health. Increasing the number of latent groups from 2 to 10 in the multinomial model improves the DIC statistics. The largest DIC improvement is observed between two and three latent groups. Other improvements in DIC between groups are relatively small and increasingly so as the number of groups increases. The ranges of the predicted TTO values (difference between the lowest and the highest value) are similar when comparing 3‐ and 10‐group models. On grounds of parsimony and to avoid having the chosen model overfit the data, the model with three groups is preferred and reported. All models have their 5, 9, and 10 parameters specifications estimated. Further details are available upon request from the authors.

**Table 4:** Estimates using all data 20‐parameter model with different slope distributions

	Normal slope		Lognormal slope		Multinomial slope	
	Coef	SD	Coef	SD	Coef	SD
Mobility						
Slight	0.057	0.008	0.051	0.009	0.027	0.004
Moderate	0.081	0.010	0.078	0.010	0.035	0.005
Severe	0.209	0.010	0.211	0.011	0.096	0.006
Unable	0.256	0.011	0.261	0.011	0.127	0.006
Self‐care						
Slight	0.058	0.008	0.058	0.008	0.023	0.004
Moderate	0.088	0.010	0.086	0.010	0.037	0.005
Severe	0.173	0.010	0.179	0.011	0.076	0.006
Unable	0.210	0.010	0.215	0.010	0.094	0.006
Usual activities						
Slight	0.051	0.009	0.048	0.008	0.023	0.004
Moderate	0.073	0.009	0.067	0.009	0.029	0.004
Severe	0.167	0.009	0.169	0.009	0.075	0.005
Unable	0.174	0.009	0.177	0.009	0.085	0.005
Pain/discomfort						
Slight	0.068	0.008	0.064	0.008	0.029	0.004
Moderate	0.088	0.009	0.090	0.010	0.039	0.005
Severe	0.262	0.010	0.268	0.011	0.128	0.007
Extreme	0.323	0.011	0.334	0.012	0.155	0.007
Anxiety/depression						
Slight	0.093	0.007	0.091	0.009	0.036	0.004
Moderate	0.122	0.009	0.123	0.010	0.048	0.005
Severe	0.295	0.009	0.301	0.010	0.132	0.006
Extreme	0.297	0.009	0.304	0.010	0.134	0.006
Variance	3.065	0.200	3.029	0.179		
P(Group1)					0.332	0.018
P(Group2)					0.388	0.020
P(Group3)					0.281	0.020
Slope (Group 1)					0.992	0.031
Slope (Group 2)					2.091	0.073
Slope (Group 3)					3.625	0.151
DIC	16732.6		16502.3		15826.5	

*Note*. DCE = discrete choice experiment; DIC = deviance information criterion; TTO = time trade‐off.

When comparing the predicted EQ‐5D index from the least severe and most severe health states based on three models that account for heterogeneity and heteroscedasticity in Table 4, we find that the prediction of health state 11211 scores at or above 0.949. This is higher than its mean observed value of 0.89, which we believe to be biased due to the asymmetry of the error distribution (see Section 2.5.3). The score for 55555 varies across models: −0.260 for the normal slope model, −0.291 for the lognormal slope model, and −0.285 for the multinomial slope model. The lower values reflect the potential of the latter two models to capture more extreme values.

The DIC is used to compare the performance between the three 20‐parameter hybrid models with different assumptions of distribution in slope. Among the three models, the lowest DIC is achieved in the multinomial slope model, that is, 15826.5. We compared two sets of estimated coefficients between the 20‐parameter multinomial slope model and 20‐parameter restricted model with TTO data treated as interval values. The coefficients are very close to each other with mean of absolute differences of 0.003. The multinomial slope model that accounts for heterogeneity and heteroscedasticity is considered as the best performing model for the data from the EQ‐5D‐5L value set for England project.

We made a number of decisions about how to interpret the data, and these were subjected to sensitivity analysis (Table 5). Column 2 reports results from our best model with data exclusion and censoring. Column 3 shows the results if we had not applied any exclusion criteria to the raw data set. Hence, there are 996 individuals included in the analysis. Health state 11211 has the highest index value of 0.949. The lowest value is reported as −0.189 for health state 55555. Columns 4 to 7 show the results without censoring at −1, without censoring at 1, without censoring at 0, and with no censoring at all, respectively. Calculations from the four sets of results in columns 4 to 7 all suggest that health state 11211 has the highest value. The lowest EQ‐5D index value is for health state 55555. The EQ‐5D index is in a range of [−0.217, 0.950] if the TTO data are not left censored at −1; [−0.277, 0.940] if TTO data are not right censored at 1; [−0.147, 0.948] if the TTO data are not censored at 0; and [−0.110, 0.938] if the TTO data are not censored at all.

**Table 5:** Effects of different interpretations of the hybrid model data

	Multinomial slope		No exclusions		No censoring at −1		No censoring at 1		No censoring at 0		No censoring at all	
	Coef	*SD*	Coef	*SD*	Coef	*SD*	Coef	*SD*	Coef	*SD*	Coef	*SD*
Mobility												
Slight	0.027	0.004	0.014	0.002	0.026	0.004	0.029	0.004	0.030	0.004	0.032	0.004
Moderate	0.035	0.005	0.016	0.002	0.032	0.004	0.035	0.005	0.038	0.005	0.037	0.004
Severe	0.096	0.006	0.043	0.004	0.087	0.006	0.095	0.005	0.097	0.006	0.092	0.005
Unable	0.127	0.006	0.055	0.005	0.111	0.006	0.124	0.006	0.122	0.007	0.114	0.006
Self‐care												
Slight	0.023	0.004	0.012	0.002	0.024	0.004	0.028	0.004	0.028	0.004	0.032	0.004
Moderate	0.037	0.005	0.016	0.002	0.036	0.004	0.037	0.005	0.041	0.005	0.040	0.004
Severe	0.076	0.006	0.034	0.003	0.069	0.005	0.076	0.005	0.078	0.006	0.075	0.005
Unable	0.094	0.006	0.041	0.004	0.086	0.005	0.096	0.005	0.091	0.005	0.088	0.005
Usual activities												
Slight	0.023	0.004	0.011	0.002	0.022	0.004	0.028	0.004	0.026	0.004	0.031	0.004
Moderate	0.029	0.004	0.013	0.002	0.028	0.004	0.031	0.004	0.034	0.005	0.035	0.004
Severe	0.075	0.005	0.032	0.003	0.069	0.005	0.076	0.005	0.078	0.005	0.076	0.005
Unable	0.085	0.005	0.034	0.003	0.075	0.005	0.086	0.005	0.083	0.005	0.080	0.005
Pain/discomfort												
Slight	0.029	0.004	0.014	0.002	0.027	0.004	0.035	0.004	0.032	0.004	0.037	0.004
Moderate	0.039	0.005	0.018	0.002	0.037	0.005	0.041	0.004	0.041	0.005	0.041	0.004
Severe	0.128	0.007	0.055	0.005	0.113	0.007	0.126	0.006	0.125	0.007	0.117	0.006
Extreme	0.155	0.007	0.066	0.006	0.137	0.007	0.157	0.007	0.148	0.008	0.140	0.006
Anxiety/depression												
Slight	0.036	0.004	0.017	0.002	0.033	0.004	0.045	0.004	0.040	0.005	0.047	0.004
Moderate	0.048	0.005	0.021	0.003	0.043	0.005	0.052	0.005	0.052	0.005	0.054	0.005
Severe	0.132	0.006	0.059	0.005	0.120	0.006	0.135	0.006	0.132	0.007	0.129	0.006
Extreme	0.134	0.006	0.059	0.005	0.122	0.006	0.137	0.006	0.134	0.007	0.130	0.006
DIC	15826.5		18098.5		15365.9		13393.4		15177.7		11959.7	

*Note*. DCE = discrete choice experiment; DIC = deviance information criterion; TTO = time trade‐off.

## 5 DISCUSSION AND CONCLUSIONS

The primary aim of this study was to develop the modelling methods to be used to produce value sets for EQ‐5D‐5L, using data generated from the newly designed elicitation protocol, comprising a different type of TTO task and with the addition of DCE data. Additionally, a number of developments in the modelling approaches were made which, compared to earlier approaches, arguably bring the models closer to the nature of the data.

The new study design combined TTO data with DCE data. The variant of lead time TTO approach that was used meant that the minimum value respondents could score was −1 without information about whether that value or a lower value represented respondents' genuine preferences. The data were seen as censored as true values were not observed. Therefore, an assumption should be made for the left tail of the distribution of the TTO data.

In contrast to the EQ‐5D‐5L value set studies published so far from the international protocol, we use both TTO and DCE data to generate a value set for England. We experimented with a number of hybrid modelling specifications that assumed a normal distribution with errors and accounting for the heteroscedasticity of TTO values between health states. We experimented with three hybrid modelling specifications that account for the heterogeneity of respondents. The assumption of normal distribution with errors is still applied. Among the three models assessing heterogeneity, we experimented with modelling specifications that assumed the slope parameter for disutility of health with a normal distribution, a lognormal distribution, and a multinomial distribution. Our results suggest that the assumptions of distributions affect the value of severe health states, as well as the predicted TTO values in particular for values where the real data points in our data set are limited. In contrast, the respondents in the Measurement and Valuation of Health (MVH) study could score TTO values as low as −39 (Dolan, 1997). In order to minimise the effect of extreme values in that study, a decision was taken to rescale the TTO values to a range of [−0.975, 1]. The data show that extreme negative values in TTO are possible. Indeed, some respondents may want to avoid—*coûte que coûte*—certain health states and their values may have great impact on the averages. The solution may be to use medians or to exclude extremes at both the lower and upper end of the scale.

By censoring at −1, the information was used that the value was either −1 or below −1. In such cases, the error distribution, which defines the left tail under −1, may have an important effect on the final estimates. Usually, normal error distributions are assumed, despite the fact that any test on the data would reject such assumption. The model estimated here, which includes heteroscedasticity, fits into this tradition. This means that if one imagines an ordered, decreasing, line of all health states, that the uncertainty surrounding each health state follows a normal distribution, which gets wider the worse the health state is.

As alternatives, we experimented with three models that assumed the slope of the imagined line differs per individual, which also implies that confidence intervals widen for lower health state values. Subsequently, we assumed that the shape of the line would follow a normal distribution, a lognormal distribution, and a multinomial distribution. When assuming a normal distribution, one has to imagine that the slope follows a normal distribution that is topped by a normal distribution surrounding each answer. This allows for a slightly thicker tail towards lower values than when assuming a heteroskedastic model. When assuming a lognormal model, one allows for much thicker tails towards negative values, but in both cases, one assumes a relatively smooth error distribution. The model that assumes a multinomial distribution of the errors is more flexible in capturing a multimodal error distribution, which is more pronounced with worsening values. And indeed, our results suggest that the choice of the distribution affects the predicted TTO values, in particular the lower values.

Respondents' opinions are truncated at the upper end of the TTO scale. We interpret the data as being censored at 1. This does not suggest that we believe the true TTO values are greater than one but rather that a combination of respondents' true TTO values plus a measurement error might be. The observed average TTO value for the mild health states is clearly very low and may not reflect the true average value for such states in the English population. Fitting the TTO data into a normal distribution with assumption of right censoring at 1 (which is not too different from taking the median) could better represent the “real” average values. It is therefore arguable that the TTO values for mild health states in the MVH study were too low, as those data are also right censored at 1.

Another aspect that has been given attention is the fact that respondents can only give 41 distinct values in the TTO data. Therefore, when a respondent scores 0.9, instead of 0.85 or 0.95, it might indicate that the true value lies between 0.875 and 0.925. Furthermore, respondents typically have a digit preference. They are more likely to provide a valuation of 0.1 than 0.05 or 0.15, for example. We used the simple correction for our heteroskedasticity model by censoring the upper end of TTO value at 0.975 rather than 1. Together with the left censoring at −0.975, this censoring exercise is also applied to the heterogeneity models, which are estimated without defining intervals for other TTO values. This characteristic of the TTO data was not recognised by the MVH study, although respondents could score more (80) unique values.

With the addition of the DCE data, we choose to combine the information into a single likelihood function, assuming that the underlying preference function that dictates the answers to the DCE comparisons also dictates the answers to the TTO questions. It should be noted that analysis of the DCE data assumes that difference in errors, or differences of opinion, follow an extreme value distribution. The difference in errors between two health states in a DCE pair follows a logistic distribution (similar to a normal distribution but with longer tails to take into account extreme values). This might be true for errors, but is unlikely to be true for differences in opinion. In the multinomial model, we identify a group of respondents who always score positive values, a group of respondents who score both positive and negative values, as well as a group of respondents with extreme values. The DCE data, in this design, are not rich enough to pick up such clear differences. Therefore, estimations that are based on DCE data only might be criticised for applying such an assumption regarding the error distributions. However, in contrast to some of the findings of modelling TTO data only, there is only one logical inconsistency in all parameters in the DCE modelling results. Also, the results from the DCE models show the general structure of the value set, that is, with small steps between the slight and moderate levels, large steps between the moderate and severe levels, and again small steps between the severe and extreme/unable levels. It is observed that the TTO data and the DCE data lead to different parameter estimates. The TTO method is arguably the closest to the decision making context where trade‐offs need to be made between length of life and quality of life. One may criticise the error distribution of the DCE model, but this may also apply to the TTO data.

The last remark refers to our decision to censor some of the data at zero. Some respondents show clear inconsistencies, scoring health state 55555 at zero and more than one other state below zero. Others used zero multiple times as their minimum value, including in the valuation of 55555, failing to prioritise between health states that follow a logical ordering. Our solution to censor these data reflects a (admittedly rather arbitrary) judgement that in such cases respondents only provided information that the value of the health state is either zero or below zero. These responses may be a consequence of the interview process, or of interviewer and/or respondent comprehension.

Another aspect that requires further justification is that we formulated the prior distributions of the level parameters to guarantee that in each dimension, the coefficients between two adjacent levels are logically consistent. Indeed, when estimated without constraint, the TTO data may suggest that the severe level is worse than unable/extreme level in the usual activities and anxiety/depression dimensions. One explanation for this may relate to the selection of the 86 health states in the TTO exercise. Additionally, we find justification of our priors by referring to the research underlying the choice of the labels in the EQ‐5D‐5L instrument (Luo, Wang, Thumboo, & Herdman, 2015).

Our study excluded respondents whose TTO data contained strong inconsistencies. We recognise that these exclusion criteria could have been extended to exclude still further data that exhibited various types of weaker inconsistencies. It is possible that the results may have been different had different levels of judgements about excluding logically inconsistent data been applied (Devlin, Hansen, Kind, & Williams, 2003). There are no agreed rules to guide researcher decisions about this. We balanced the desire to base modelling on plausible data with a desire to minimise exclusions from the sample, both to protect the representativeness of the sample and to guard against excluding data that are inconvenient but not entirely implausible.

In the end, we find that a model that splits the population into three groups of preference “types” appeals both intuitively and in terms of the statistical performance of the models. The result captures the idea that the groups have different attitudes towards death when making trade‐offs between quality and length of life. This may help people identify themselves when considering the outcomes of a value function. The final model is not one model for all; rather, it is a compromise of different opinions, statistics, and trying to capture the opinions of a nation with different—sometimes very different—opinions.

## ETHICAL STATEMENT

The study was given approval by the research ethics committee of the University of Sheffield's School of Health and Related Research.

## ORIGINAL PUBLICATION

This manuscript is not being submitted for publication elsewhere at the same time.

## FUNDING SOURCE

This study was funded by a Department of Health Policy Research Programme grant (National Institute for Health Research PRP 070/0073). Additional funding and technical support was provided by the EuroQol Research Foundation.

## CONFLICTS OF INTEREST

YF, NJD and KKS are employees of the Office of Health Economics, a registered charity which receives funding from a variety of sources, including the Association of the British Pharmaceutical Industry. All authors are members of the EuroQol Group.

## Supporting information

Appendix S1. Background characteristics of the sample
