# Supplementary material for: New methods for modelling EQ‐5D‐5L value sets: An application to English data
Source: Health Econ. 2017 Aug 18;27(1):23–38. doi: 10.1002/hec.3560 (PMC5836982; doi:10.1002/hec.3560)
Supplement: Supplementary file 1 — Appendix S1. Background characteristics of the sample [file HEC-27-23-s001.docx]

**Appendix I. Background characteristics of the sample**

|  | **All participants (n=996)**^i^ | **After exclusions (n=912)**^i^ | **General population**^ii^ |
| --- | --- | --- | --- |
|  | **N (%)** | **N (%)** | **%** |
| Age  18-29  30-44  45-59  60-74  75+ | 113 (11.3)  298 (29.9)  250 (25.1)  207 (20.8)  128 (12.9) | 105 (11.5)  270 (29.6)  227 (24.9)  191 (20.9)  119 (13.0) | 20.7%  26.3%  24.7%  18.5%  9.9% |
| Gender  Male  Female | 405 (40.7)  591 (59.3) | 372 (40.8)  540 (59.2) | 49.2%  50.8% |
| Economic activity  Employed or self-employed  Retired  Student  Looking after home or family  Long-term sick or disabled  Other / none of the above | 504 (51.2)  278 (28.2)  20 (2.0)  83 (8.4)  48 (4.9)  52 (5.3) | 463 (50.8)  256 (28.1)  19 (2.1)  73 (8.0)  42 (4.6)  47 (5.2) | 59.4%  13.1%  8.8%  4.2%  3.9%  10.6% |
| Marital status  Never Married  Married  Same-sex civil partnership  Separated ^iii^  Divorced  Widowed ^iv^  Prefer not to say | 238 (24.2)  466 (47.3)  2 (0.2)  37 (3.8)  131 (13.3)  107 (10.9)  4 (0.4) | 225 (24.7)  434 (47.6)  2 (0.2)  32 (3.5)  119 (13.0)  99 (10.9)  1 (0.1) | 34.6%  46.6%  0.2%  2.7%  9.0%  6.9%  N/A |
| Religion  Christian  Any other religion  No religion  Religion not stated | 636 (64.6)  60 (6.1)  281 (28.5)  8 (0.8) | 575 (63.9)  53 (5.9)  266 (29.6)  6 (0.7) | 59.4%  8.7%  24.7%  7.2% |
| Ethnicity  White  Any other ethnic group  Prefer not to say | 900 (91.4)  82 (8.3)  3 (0.3) | 832 (92.4)  67 (7.4)  1 (0.1) | 85.4%  14.6%  N/A |
| Day-to-day limitations due to health problem or disability  Limited a lot  Limited a little  Not limited | 111 (11.3)  158 (16.0)  716 (72.7) | 95 (10.6)  144 (16.0)  661 (73.4) | 5.6%^v^  7.1%^v^  87.3%^v^ |
| Education  Degree  No degree | 211 (21.4)  774 (78.6) | 201 (22.3)  699 (77.7) | N/A |
| Main language spoken  English  Any other language | 920 (93.4)  65 (6.6) | 847 (94.1)  53 (5.9) | N/A |
| Responsibility for children  Yes  No | 350 (35.5)  635 (64.5) | 314 (34.9)  586 (65.1) | N/A |
| Experience of serious illness  In self  In family  In caring for others | 330 (33.1)  692 (69.5)  416 (41.8) | 297 (32.6)  636 (69.7)  385 (42.2) | N/A |
| Self-rated health using EQ-5D-5L  11111  Any other health state | 474 (47.6)  522 (52.4) | 437 (47.9)  475 (52.1) | N/A |
| Self-rated health using EQ-VAS  <80  80-89  90-99  100 | 334 (33.5)  256 (25.7)  337 (33.8)  69 (6.9) | 298 (32.7)  241 (26.4)  306 (33.6)  67 (7.3) | N/A |

^i^ Data on economic activity, marital status, religion, ethnicity, day-to-day limitations, main language and responsibility for children unavailable for a minority of participants

^ii^ Based on results of the 2011 UK Census (Office for National Statistics, 2011), where available; N/A indicates that a directly comparable question was not included in the 2011 Census

^iii^ Comprises individuals who are separated but still legally married or in a same-sex civil partnership

^iv^ Includes individuals who are the surviving partner from a same-sex civil partnership

^v^ Census data reported here refers to individuals aged 16-64 only
